# Neonatal Meningitis: Overcoming Challenges in Diagnosis, Prognosis, and Treatment with Omics

**DOI:** 10.3389/fped.2017.00139

**Published:** 2017-06-16

**Authors:** Scott M. Gordon, Lakshmi Srinivasan, Mary Catherine Harris

**Affiliations:** ^1^Division of Neonatology, Children’s Hospital of Philadelphia, Philadelphia, PA, United States; ^2^Division of Neonatology, Children’s Hospital of Philadelphia, Perelman School of Medicine, Philadelphia, PA, United States

**Keywords:** meningitis, neonatology, cytokines, metabolomics, proteomics, transcriptomics

## Abstract

Neonatal meningitis is a devastating condition. Prognosis has not improved in decades, despite the advent of improved antimicrobial therapy and heightened index of suspicion among clinicians caring for affected infants. One in ten infants die from meningitis, and up to half of survivors develop significant lifelong complications, including seizures, impaired hearing and vision, and delayed or arrested development of such basic skills as talking and walking. At present, it is not possible to predict which infants will suffer poor outcomes. Early treatment is critical to promote more favorable outcomes, though diagnosis of meningitis in infants is technically challenging, time-intensive, and invasive. Profound neuronal injury has long been described in the setting of neonatal meningitis, as has elevated levels of many pro- and anti-inflammatory cytokines. Mechanisms of the host immune response that drive clearance of the offending organism and underlie brain injury due to meningitis are not well understood, however. In this review, we will discuss challenges in diagnosis, prognosis, and treatment of neonatal meningitis. We will highlight transcriptomic, proteomic, and metabolomic data that contribute to suggested mechanisms of inflammation and brain injury in this setting with a view toward fruitful areas for future investigation.

## Introduction

Meningitis is a life-threatening disease, affecting 0.1–0.4 neonates per 1,000 live births, with a higher incidence in preterm and chronically hospitalized infants (1, 2). Approximately 10% of affected infants die, and 20–50% of survivors develop seizures, cognitive deficiencies, motor abnormalities, and hearing and visual impairments (3). Despite declines in mortality, morbidity has not improved since the 1970s.

Rapid initiation of appropriate broad-spectrum antimicrobial therapy in response to suspected neonatal meningitis is critical to optimize outcomes (4). Empiric therapy often chosen in the setting of suspected early-onset sepsis/meningitis ensures coverage of Group B Streptococcus (GBS), *Listeria monocytogenes*, and Gram-negative organisms, such as *Escherichia coli* (4, 5). Suspected late-onset infections are typically treated with even broader antimicrobial therapy to cover additional organisms in the nosocomial environment, including *Pseudomonas aeruginosa* and methicillin-resistant *Staphylococcus aureus*. Antibiotic therapy can only be narrowed if bacterial cultures reveal an offending organism, which hinges on swift, successful performance of a technically challenging, and sometimes risky, lumbar puncture (LP) prior to administration of antibiotics.

At a minimum, recommended therapy for meningitis lasts several weeks and may be longer based on organism or more extensive disease, such as ventriculitis or abscess formation (5). Infants with negative cultures despite a suspicion of meningitis are often managed conservatively and receive a full course of antibiotic therapy. In neonates, long-term antibiotic therapy is associated with emergence of resistant bacteria, superimposed fungal infections, and increased risk of necrotizing enterocolitis (6). Additional risks associated with long-term antibiotic therapy include need for central vascular access and its attendant complications (7). Thus, infants may sustain harm not only from meningitis but also from associated interventions.

Prognosis in the setting of neonatal meningitis is also fraught with difficulty. While outcomes are influenced by time to diagnosis and therapy, prognosis may also relate to virulence of the infecting pathogen. With our current understanding of meningitis, however, it is not possible to predict which infants will die, which infants will survive with disabilities, and which infants will survive with a normal neurodevelopmental outcome. Indeed, many infants still die, and many survivors still sustain lifelong morbidities, despite rapid initiation of appropriate antimicrobial therapy (8, 9).

Several adjuncts to antibiotic therapy have been proposed and tested to improve poor outcomes associated with meningitis. Steroid administration has not been shown to be beneficial as an adjunct to antimicrobial therapy in neonatal bacterial meningitis (10). While there is evidence for improved neurologic and auditory outcomes in pediatric *Hemophilus influenzae* B meningitis following steroid therapy, this pathogen is not a frequent cause of meningitis in neonates (11). The data surrounding benefit of steroids in pediatric or neonatal meningitis secondary to GBS, *E. coli, Streptococcus pneumonia*, and *Neisseria meningitidis* is unclear or poor, while long-term risks of exposing neonates to steroids are incompletely understood. Therefore, steroids are not recommended as adjunctive therapy in neonates evaluated for meningitis, unless there is strong suspicion for *H. influenzae* infection based on Gram stain or culture results. Trials of other adjunctive therapies, such as intravenous immunoglobulin and granulocyte-monocyte colony-stimulating factor, have also shown disappointing results.

To enhance outcomes, we must gain deeper insights into the pathophysiology of meningitis to identify diagnostic and prognostic tools and therapies that facilitate bacterial clearance but limit deleterious immune-mediated damage to brain tissue. Herein, we review challenges associated with the rapid and accurate diagnosis of neonatal meningitis. We discuss how a variety of large-scale datasets have extended our understanding of the host response to meningitis. Finally, we integrate the findings into a model to highlight new avenues for basic and translational investigations into the key immunologic pathways active in the setting of neonatal meningitis.

## Diagnostic Challenges in Neonatal Meningitis

Culture of cerebrospinal fluid (CSF) is the traditional gold standard for diagnosis of bacterial meningitis. However, deciding when to perform LP to obtain and analyze CSF is challenging. Factors complicating this decision include the non-specific signs and symptoms of meningitis in the infant, cardiorespiratory instability that may preclude positioning of an infant for LP, and considerable practice variation (12–15). Meningitis is estimated to occur in approximately 1–2% of suspected cases of sepsis within the first 72 h of life, or early-onset sepsis, though the risk is limited almost entirely to symptomatic infants (16). The American Academy of Pediatrics policy statement on suspected or proven early-onset bacterial sepsis supports performing LP as part of the sepsis evaluation of the neonate with symptoms concerning for early-onset sepsis, but recommends a more limited evaluation in the asymptomatic neonate with sepsis risk factors (17). In contrast to early-onset sepsis, evaluations for late-onset sepsis (after the first 72 h of life) are almost always performed in response to concerning signs and symptoms. Several studies have noted discordance between blood culture and CSF culture results, with negative blood cultures in up to a third of infants with bacterial meningitis, highlighting that this diagnosis could be missed if LP is deferred or not performed (13–15). These findings strongly support obtaining CSF *via* LP prior to antibiotic administration in neonates evaluated for late-onset sepsis. In infants with positive blood cultures, an LP is essential to guide duration of therapy and provide prognostic information.

Interpretation of CSF results is frequently problematic. If LP is delayed, and infants are exposed to empiric broad-spectrum antibiotics, clinical yield of bacterial culture of CSF can be compromised (18–20). In these situations, clinicians rely on interpretation of CSF parameters, such as cell count, glucose, and protein levels to presumptively diagnose meningitis. However, there is considerable overlap in laboratory evaluation of CSF between groups of infected and uninfected infants, leading to difficulty in establishing cutoff levels that possess sufficient sensitivity and specificity for the diagnosis of bacterial meningitis (14, 15). Many other factors influence interpretation of these values in neonates, including gestational age, postnatal age, and trauma sustained during LP causing contamination of CSF with blood (12, 14, 15, 18, 21).

Furthermore, diagnostic markers may differ depending on the gestational maturity of the infant and may confound analyses unless proper controls matched for gestational age are included. Additional studies are warranted to understand how these markers vary during normal development and to better inform how they vary in the context of neonatal meningitis. Indeed, many studies have highlighted differences in the immune system of newborns and infants relative to that of children and adults (22, 23). The premature infant may be additionally immune-compromised compared to full-term infants, due, in part, to deficiencies in innate and adaptive immune system (22). Clinically, these deficiencies manifest as increased risk for coagulase-negative *Staphylococcus* spp., *Staphylococcus aureus*, and *Candida* spp. Of note, however, in an investigation of infants with sepsis, Smith et al. found no significant differences in key genes activated or repressed in the blood of infants of varying gestational ages (24). These data suggest that some key immune pathways activated upon serious bacterial infection may be similar between the preterm and the full-term infant.

## Pathogen-Based Evaluation of CSF

A variety of approaches have been examined to improve the rapidity and fidelity of diagnosis of neonatal bacterial meningitis over conventional methods. These tests can be categorized broadly as: (1) identification of microbial signatures in CSF and (2) detection of a host response signature pathognomonic of sepsis and meningitis. There is much interest in development of bacterial nucleic acid-based polymerase chain reaction (PCR) assays for detection of common pathogens implicated in sepsis and meningitis (25). These tests have the potential advantage of rapid turnaround time compared to conventional microbiologic culture methods. Further, PCR-based testing has the ability to identify small amounts of nucleic acid signatures from non-viable bacteria, thereby improving diagnostic yield, especially in situations with low bacterial load, such as following antibiotic pretreatment (26). Investigators have studied conventional and real-time PCR methods, and several have employed broad-based bacterial PCR techniques directed against the 16S ribosomal RNA subunit that is conserved across bacterial species. However, in recent years, greater technical success has been noted with multiplex PCRs targeted to several common pathogens implicated in meningitis (27–29). Boriskin et al. reported the use of a focused microarray to detect and distinguish known genomic sequences of 13 viruses causing meningitis (30). Ben and colleagues employed a similar type of array to detect sequences of 20 common bacteria implicated in meningitis (31). Recently, a multiplex PCR assay has been approved by the FDA for detection of 14 pathogens, including *Escherichia coli* K1, *Streptococcus agalactiae, Listeria monocytogenes*, and *Haemophilus influenzae* (28, 29). Nonetheless, additional testing is required to determine the reliability of CSF PCR tests when compared with culture results in the management of bacterial meningitis in the clinical setting. To date, bacterial tests of sources other than CSF have shown disappointingly poor accuracy in diagnosis of meningitis (32).

## Cytokine-Based Evaluation of CSF

The second approach to developing diagnostic tests is based on the premise that serious infections such as meningitis elicit a specific host immune and/or metabolic response that could represent a “host signature” pathognomonic of infection (33–36). Several studies of pediatric and neonatal meningitis have evaluated cytokine levels and other candidate biomarkers in CSF for their diagnostic utility (Table 1). In a prospective cohort of infants with suspected bacterial meningitis, Srinivasan et al. noted that interleukin-6 and interleukin-10 possessed the best area under the curve (AUC) in receiver operating characteristic analyses of multiple cytokines [Table 1 and Ref. (33)]. In a study of 140 subjects with pediatric meningitis, Ye et al. identified interleukin-6 alone and the ratio of CSF/blood interleukin-6 as useful diagnostic markers [Table 1 and Ref. (37)]. However, not all investigations of interleukin-6 have replicated this success. Mukai and colleagues noted poorer diagnostic accuracy with interleukin-6 compared to tumor necrosis factor-α, and Hsieh’s group demonstrated that interleukin-6 had good sensitivity but poorer specificity than interleukin-12 [Table 1 and Ref. (38, 39)]. Prasad et al. also noted that tumor necrosis factor-α and interleukin-8 provided excellent ability to discriminate bacterial meningitis, and outperformed interleukin-6, in their cohort of 87 pediatric patients [Table 1 and Ref. (40)]. Ours and an additional group concluded that, while tumor necrosis factor-α had good specificity for bacterial meningitis in infants and children, sensitivity was less promising [Table 1 and Ref. (41, 42)]. Challenges noted by investigators include the short half-life of many cytokines with only transient elevations in CSF in the setting of bacterial meningitis. The timing of LP in relation to onset of illness, therefore, becomes a crucial factor in interpretation of levels. As noted previously, however, it is often a challenge to obtain LPs early in the course of illness in unstable infants.

**Table 1:** Studies investigating diagnostic accuracy of cerebrospinal fluid (CSF) cytokines in pediatric and neonatal meningitis.

Reference	Study population	Biomarkers evaluated	Findings
Srinivasan et al. (33)	Overall: 684 patients <6 months Bacterial meningitis: *n* = 11 Not meningitis: *n* = 151 Indeterminate (antibiotic pretreated): *n* = 513	CSF levels of TNF-α, IL-1, IL-6, IL-8, IL-10, IL-12	IL-6 and IL-10 had best area under the curve (AUC) when bacterial meningitis was compared with controls; some indeterminate infants had cytokine patterns similar to infants with bacterial meningitis TNF-α: AUC 0.88 IL-1: AUC 0.86 IL-6: AUC 0.91 IL-8: AUC 0.89 IL-10: AUC 0.91 IL-12: AUC 0.63

Ye et al. (37)	Overall: 814 patients <18 years Bacterial meningitis: *n* = 140 [derivation cohort (DC): *n* = 71; validation cohort (VC): *n* = 69] Healthy controls: *n* = 180 Viral encephalitis: *n* = 182 Epilepsy: *n* = 146 Febrile convulsions: *n* = 166	CSF levels of IL-6, IL-10 CSF/blood ratios of IL-6 and IL-10	Bacterial meningitis versus all others IL-6: AUC: 0.988 (DC); 0.985 (VC) IL-10: AUC 0.949 (DC); 0.938 (VC) CSF/blood IL-6 ratio: 0.995 (DC); 0.993 (VC) CSF/blood IL-10 ratio: 0.924 (DC); 0.912 (VC)

Prasad et al. (40)	Overall: 87 patients <14 years Bacterial meningitis: *n* = 57 Viral meningitis: *n* = 15 Not meningitis: *n* = 15	CSF levels of TNF-α, IL-6, IL-8	Bacterial meningitis versus controls TNF-α: AUC: 1 IL-6: AUC: 0.947 IL-8: AUC: 1 Bacterial meningitis versus viral meningitis TNF-α: AUC: 0.961 IL-6: AUC: 0.853 IL-8: AUC: 0.941

Hsieh et al. (39)	Overall: 95 patients <15 years Bacterial meningitis: *n* = 12 Aseptic meningitis: *n* = 41 Not meningitis: *n* = 42	CSF levels of IL-6, IL-12	IL-6: Sensitivity: 96%, specificity: 51% IL-12: Sensitivity 96%, specificity 75%

Mukai et al. (38)	Overall: 35 patients <12 years Bacterial meningitis: *n* = 6 Aseptic meningitis: *n* = 13 Not meningitis: *n* = 16	CSF levels of TNF-α, IL-6	TNF-α was detected in all bacterial meningitis cases and in 84.6% of the children with aseptic meningitis IL-6 did not enable differentiation between bacterial and aseptic infection

Tang et al. (42)	Overall: 171 specimens from 144 patients <14 years Bacterial meningitis: *n* = 23 Aseptic meningitis: *n* = 26 Not meningitis: *n* = 95	CSF levels of IL-1β, TNF-α	IL-1β: Sensitivity 78%, specificity 96% TNF-α: Sensitivity 74%, Specificity 81%

Dulkerian et al. (41)	Overall: 62 patients <6 months Bacterial meningitis: *n* = 20 Aseptic meningitis: *n* = 22 Not meningitis: *n* = 20	CSF and plasma levels of IL-6, TNF	IL-6 (CSF): Sensitivity 100%, NPV 100% TNF (CSF): Sensitivity 60%, specificity 100%

Biomarkers, or combinations of biomarkers, that demonstrate not only early but sustained elevations over the course of infection may be better candidates for diagnostic tests. Alterations in damage-associated molecular patterns (DAMPs) that aid in perpetuation of the inflammatory response may provide more stable measurement targets. S100-family proteins are DAMPs associated with innate immune activation (43). S100B showed early promise as a biomarker for meningitis due to its concentration in astrocytes and glia (44, 45). Further investigations, however, revealed that S100B in serum or CSF exhibited suboptimal sensitivity and specificity for bacterial meningitis. The highest, but still inconsistent, levels of S100B in CSF and serum were seen in viral encephalitis or in bacterial meningitis complicated by ventriculitis or obvious parenchymal abnormalities on brain imaging (44, 46). While many candidate cytokine and immunologic markers have shown promise as diagnostic tools in small studies, some have demonstrated conflicting findings across studies (Table 1). Thus, such markers have not been accepted into clinical practice as adjunctive tests for diagnosis of meningitis. These data emphasize the need for novel investigations into other classes of potential biomarkers to enhance diagnosis of meningitis beyond our current capability with clinical judgment and classical laboratory parameters.

## Omics to Extend Knowledge of Bacterial Meningitis

Recently, investigators have embarked on exploratory approaches, taking advantage of advanced “omics” technologies to more deeply understand serious bacterial infection in infants, children, and adults (34–36, 47). Omics denotes the comprehensive investigation of any family of biologic molecules, including DNA (genomics), RNA transcripts (transcriptomics), proteins (proteomics), and metabolites (metabolomics), among others. In other words, omics are used to interrogate hundreds to thousands of molecules simultaneously from a body fluid or tissue, promoting generation of pathways or networks of molecules associated with presence or absence of a disease state, such as infection. Omics approaches can provide new insights into diagnosis, pathophysiology, risk stratification and prognostication, and potential therapeutic targets (48). Systems-level investigations into host–pathogen interactions involving many cells types have informed countless translational studies of complex human diseases. Bacterial meningitis is a complex infectious disease amenable to omics analyses to generate new hypotheses into mechanisms underlying inflammation and brain injury. A common obstacle to rigorous mechanistic studies of many human disorders, use of primary human tissue to study meningitis is generally not possible. CSF is an attractive source of information, as it communicates directly or indirectly with brain-resident cells, such as neurons, astrocytes, glia, and the blood–brain barrier. CSF is also in direct contact with infiltrating cells of the immune system recruited to the brain space in the setting of meningitis. Thus, CSF contains hosts of transcripts, proteins, and metabolites that, together, can paint a clearer picture of a disease process whose morbidity and mortality remain unacceptably high despite early recognition, definitive treatment with appropriate antibiotic therapy, and intensive care. Herein, we note advances made with model systems of human meningitis, but we will focus on transcriptomic, proteomic, and metabolomic work performed in human patients with bacterial meningitis.

## Transcriptomics

Transcriptomics reveals genome-wide changes in gene expression by individual cells or populations of cells and often forms the backbone for gene-targeted analyses in animal models. Transcriptomics has evolved as an increasingly affordable and fruitful analysis, thanks in large part to several large, well-curated databases capable of organizing results into networks of known pathways. Numerous transcriptomic analyses in model systems of bacterial meningitis have utilized cultured human brain microvascular endothelial cells and cultured primary meningioma cells (49, 50). When cultured appropriately, such cells can recapitulate the blood–brain barrier *in vitro*. Both systems have been used to understand changes in host gene expression occurring upon contact with bacterial pathogens, such as *N. meningitidis* (51–53).

Animal models of meningitis have also provided great insight into the pathophysiology of neonatal meningitis, dating back to the first *in vivo* model in piglets to show disruption of the blood–brain barrier (54). Currently, rodent models of neonatal meningitis are often used to model human bacterial meningitis (55–58). One study by Coimbra and colleagues used a high-fidelity infant rat model of pneumococcal meningitis to separately profile the transcriptomes of the cortex and hippocampus in the early and late phases of disease (59). This model faithfully replicates much of the pathophysiology found in humans insofar as infant rats suffer similar cortical ischemia with resultant necrosis and hippocampal apoptosis as do human neonates who contract bacterial meningitis (60). Many genes associated type 1 inflammation and toll-like receptor activation, such as interleukin-6, interleukin-18, and STAT1, were increased in both the cortex and hippocampus of infant rats after induction of meningitis (59). Consistent with the distinct mechanisms of injury in the cortex and hippocampus, however, Coimbra et al. found differential regulation of genes associated with toll-like receptor signaling and innate immune activation, including CD14 and tumor necrosis factor-α, in each brain region. Supporting histologic findings, pro-apoptotic transcripts were upregulated selectively in the hippocampus, while increased expression of tropomyosin selectively in the cortex may reflect excess vasoconstriction and ischemia.

Another intriguing set of transcripts found to be upregulated in the cortex and hippocampus of infant rats with meningitis were several associated with complement activation, including C1q, C3, and C4 (59). In addition to a canonical role for complement in bacterial clearance, recent work has highlighted an additional role for complement in crosstalk with astrocytes and microglia to eliminate neuronal synapses (61–63). Indeed, a transcript indicative of activation of astrocytes (glial fibrillary acidic protein, or GFAP) was similarly upregulated in both the cortex and hippocampus of infant rats with meningitis. While clearance of synapses in the setting of bacterial meningitis has not been formally addressed, it is tempting to speculate that this may represent a critical mechanism of brain injury. Altogether, these data highlight the critical importance of animal models to our understanding of bacterial meningitis in human neonates. They also shed light on potential therapeutic targets as we gain greater knowledge of appropriate and maladaptive immune responses to bacterial meningitis.

While the transcriptome of brain tissue or CSF has not been examined in humans with meningitis, profiling of the transcriptome of whole blood in human patients with bacterial meningitis was performed by Lill and colleagues (64). Although the bulk of their study cohort was composed of adult patients, three neonates were included in the analysis: two with GBS meningitis and one with *E. coli* meningitis. Of note, clustering analysis of their data revealed that the neonates with meningitis had distinct whole blood transcriptomic profiles from the adults with meningitis. While adults with meningitis had a distinct whole blood transcriptome compared to adults without infection, the whole blood transcriptome of two out of three neonates most closely resembled that of control adults. This emphasizes the need for infant-specific studies of meningitis, as the neonatal and adult host responses to infection vary substantially (23, 65).

In the setting of bacterial meningitis, the most significantly changed individual and networks of transcripts in the blood were those associated with innate and adaptive immunity (64). Interestingly, type 2 immunity-associated genes, including interleukin-5 receptor, Fc-epsilon receptors, and markers of mast cell activation, were among the most upregulated in the blood of patients with bacterial meningitis relative to controls. These data support findings that type 2 immune responses are induced in humans and animal models with serious bacterial infection (66). In fact, type 2 immune signaling to macrophages drives better outcomes in animal models of sepsis and is associated with reduced severity of disease in humans with sepsis (66, 67). The data also are congruent with work to profile the inflammatory cytokine milieu in CSF of infants and children with bacterial meningitis, described above, showing type 2 cytokines interleukin-4 and interleukin-13 significantly upregulated in the CSF of infants and children with bacterial meningitis compared to controls (33, 68). Future studies will be needed to understand the significance of type 2 immunity in the setting of bacterial meningitis, especially in neonates who often exhibit blunted type 1 immune responses (65).

Notably, the study by Lill and colleagues (64) represents a first step to detect a signature of bacterial meningitis in the blood, outside of the brain space. Such lines of investigation may drive discovery and development of minimally invasive tools to diagnose bacterial meningitis, one day obviating the need for LP.

## Proteomics

One caveat of transcriptomics is that some transcripts may not be translated into protein due to posttranscriptional modifications or regulation by non-coding RNA species. Transcriptomics also has limited clinical utility in the intensive care unit, as detection of mRNA transcripts can be expensive and time consuming. Proteomics offers the promise of novel biomarker discovery that may easily translate to clinical applications. Jesse and colleagues (69) applied the first large-scale proteomic approach to the study of bacterial and viral meningitis in adults. Analyzing CSF from adult patients with fluorescent two-dimensional difference gel electrophoresis, the authors aimed to find a protein signature of bacterial meningitis that differentiates it from viral meningitis and from controls. Six proteins were identified as candidate markers of bacterial meningitis in a pilot experiment and were subsequently validated in a larger cohort of patients. Interestingly, GFAP was one such protein marker specific to bacterial meningitis that was also identified by Coimbra et al. as an increased transcript in the setting of bacterial meningitis in infant rats (59). Activation of astrocytes and expression of GFAP can be induced by pro-inflammatory cytokines, such as interleukin-6 (70). Another protein marker of bacterial meningitis identified by Jesse et al. was Prostaglandin-H2 D-isomerase or prostaglandin D synthase (69). Recently, prostaglandin D synthase was implicated as a marker of potent T helper 2 cells that strongly elaborate type 2 cytokines, including interleukin-4, interleukin-5, and interleukin-13 (71). These data invoke the transcriptomic findings of Lill and colleagues, who showed increased levels of transcripts associated with type 2 immunity in the serum of patients with bacterial meningitis (64). As evidenced by this work, multiple omics modalities can be combined and used to link data across multiple studies and disciplines.

Using similar methodology to that of Jesse et al., Goonetilleke and colleagues performed comparative two-dimensional polyacrylamide gel electrophoresis to characterize the proteome of CSF from adult humans with pneumococcal meningitis (72). The focus of their analysis was to identify markers differentially regulated in survival versus death. Among the proteins relevant to the immune response, complement C3 was decreased in non-survivors, suggesting impaired complement-mediated bacterial clearance in the brain space or, possibly, impaired clearance of injured neurons by astrocytes and microglia (61–63), as discussed above. Another interesting finding was an increase in chitotriosidase in CSF of non-survivors. Chitotriosidase is associated with activation of macrophages, especially those exposed to type 2 inflammatory cytokines (73). As discussed above, there are several pieces of data supporting robust type 2 inflammation in bacterial meningitis, but whether excessive type 2 immune bias in the setting of bacterial meningitis is maladaptive and associated with poor outcomes remains to be investigated. The data of Goonetilleke et al. should be interpreted with some caution, as all patients with meningitis were coinfected with HIV in this study, but the interesting findings begin to address a critical need to predict not only diagnosis but also prognosis of bacterial meningitis. This is especially important in the neonatal population, whose potential disabilities due to meningitis are numerous and would benefit from early subspecialist involvement and intervention.

Cordeiro et al. presented the most recent proteomic data in the setting of meningitis, with a goal to differentiate among control subjects, those with pneumococcal meningitis, those with meningococcal meningitis, and those with enteroviral meningitis (74). The cohort consisted of adults and children, and CSF was analyzed using two-dimensional protein gel electrophoresis. The authors propose an algorithm to predict bacterial meningitis based on elevated apolipoprotein A1, also found to be elevated in bacterial meningitis by Song and colleagues (75), and complement C3, which participates in clearance of encapsulated microbes and in clearance of neuronal synapses (61–63). Once classified as bacterial meningitis, kininogen-1 was identified to discriminate between pneumococcal and meningococcal disease, though kininogen-1 is a pleiotropic effector molecule with possible, but poorly defined, effects on pro-inflammatory cytokine signaling, neurotransmission, and blood–brain barrier function (74). Overall, proteomic approaches to the study of bacterial meningitis are powerful and may lay the groundwork for future translational studies and clinical assays to improve rapidity and fidelity of diagnosis. While pediatric subjects were more represented in the study by Cordeiro and colleagues, there remains an absence of work dedicated to comprehensively addressing the proteome of neonates with bacterial meningitis.

## Metabolomics

While approaches to simultaneously evaluate multitudes of metabolites have become available only recently, metabolic analysis of CSF is nearly a century old (76). Consistent with presumed cellular stress and high metabolic demand, elevated levels of lactate and decreased levels of glucose were among the first markers to be associated with meningitis. Since the original studies, lactate has been well validated as a highly accurate marker of bacterial meningitis (77). Additional investigations into the CSF metabolome of patients with meningitis have been performed, with a goal of discovering novel biomarkers of disease and uncovering aberrantly regulated pathways contributing to maladaptive inflammation and brain injury. Metabolomics offers an advantage over proteomics and transcriptomics in that metabolites are the final products of active enzymatic reactions in a cell or group of cells. In other words, metabolomics most accurately reflects the true cellular phenotype without having to consider posttranscriptional or posttranslational modifications that may add layers of complexity to transcriptomic or proteomic datasets, respectively. Challenges to pursuing metabolomic work, however, include expense, non-standardized modalities to acquire data, and still-evolving databases with which to compare and contextualize results.

Several studies of CSF in humans with bacterial meningitis employ proton nuclear magnetic resonance (NMR) spectroscopy to study a varied number of metabolites (78–80). Using NMR spectroscopy, Subramanian et al. investigated whether metabolites could distinguish CSF of children with tuberculous meningitis from CSF of children with non-tuberculous bacterial meningitis, viral meningitis, or no meningitis (79). One fascinating finding was detection of the metabolite cyclopropane specifically in CSF from children with tuberculous meningitis (79). Modifications to mycolic acids with cyclopropane in the cell wall of *M. tuberculosis* are critical for virulence (81). This piece of data highlights the potential of metabolomics to identify key pathways relevant to diagnosis and pathophysiology of bacterial meningitis.

In the study by Subramanian et al., other metabolites significantly elevated in CSF of those with bacterial meningitis relative to viral meningitis or controls included urea, creatine, alanine, citrate, pyruvate, acetoacetate, and beta-hydoxybutyrate (79). These data, particularly elevations in acetoacetate and beta-hydroxybutyrate, suggest increased ketosis and an increase in circulating free fatty acids that are subsequently metabolized into ketone bodies. Interestingly, poor outcomes in a cohort of adults with sepsis have been associated with increased levels of fatty acids in the serum (82). Further underscoring the importance of fatty acid oxidation in the immune response to infection, pro-inflammatory responses have been linked to activation of glycolytic pathways, while oxidation of fatty acids and ketosis has been linked to anti-inflammatory pathways in neonates with sepsis (22, 24). Altogether, these data may reflect inappropriately blunted immunity that may predispose to poor outcomes after serious bacterial infection, including sepsis and meningitis.

Overall, Subramanian et al. quantified twelve metabolites but distinguished non-tuberculous bacterial meningitis from controls with sensitivity and specificity of only 74 and 67%, respectively. When clinical variables were also considered, however, the model’s sensitivity and specificity rose to nearly 100%. These data speak to the value of combining multiple modalities in omics research, an approach recently used to create a high-fidelity clinical-metabolomic model of outcomes after bacterial sepsis in adult humans (82).

Coen et al. evaluated the CSF metabolome of mainly adult humans with bacterial, fungal, and viral meningitis, as compared to that of control adults and those with indwelling neurosurgical hardware (80). Of note, the cohort did include two infants with *S. agalactiae* (Group B Strep) meningitis. The group addressed a critical question relevant to the population in the NICU, as a small but significant proportion of infants born extremely prematurely will develop intraventricular hemorrhage and, subsequently, posthemorrhagic hydrocephalus. Using NMR spectroscopy to ascertain metabolic composition of CSF, Coen and colleagues were able to distinguish patients with bacterial meningitis from those with viral meningitis and those with no meningitis but with indwelling hardware. The most “influential” metabolites used to separate experimental groups were glucose and lactate, with additional contribution from beta-hydroxybutyrate, pyruvate, acetate, acetone, isoleucine, leucine, and valine. Of note, branched-chain amino acids, especially valine, have been shown to promote maturation, antigen presentation capability, and cytokine production of innate immune cells (83). The investigators were not able to distinguish CSF infected with Gram-positive organisms from CSF infected with Gram-negative organisms (80), in contrast to the findings of Lill and colleagues, who found unique changes in the plasma transcriptome of patients with bacterial meningitis due to pneumococcus relative to those with meningitis due to other bacterial organisms (64). This is perhaps due to small sample size, though it is feasible that the active metabolic pathways and mechanisms of cellular injury contributing to the CSF metabolome may not vary substantially among different microorganisms.

Although expensive, mass spectrometry-based approaches to metabolomics are alternatives to NMR spectroscopy and are capable of characterizing hundreds of metabolites from body fluids and tissues (84). No studies utilizing mass spectrometry-based metabolomics have been performed in patients, especially infants, with bacterial meningitis. To fill this gap in knowledge and complement known transcriptomic and proteomic data, it will be necessary to more comprehensively investigate the metabolome of CSF in model organisms and in human infants with bacterial meningitis.

## Conclusion

**Figure 1:**
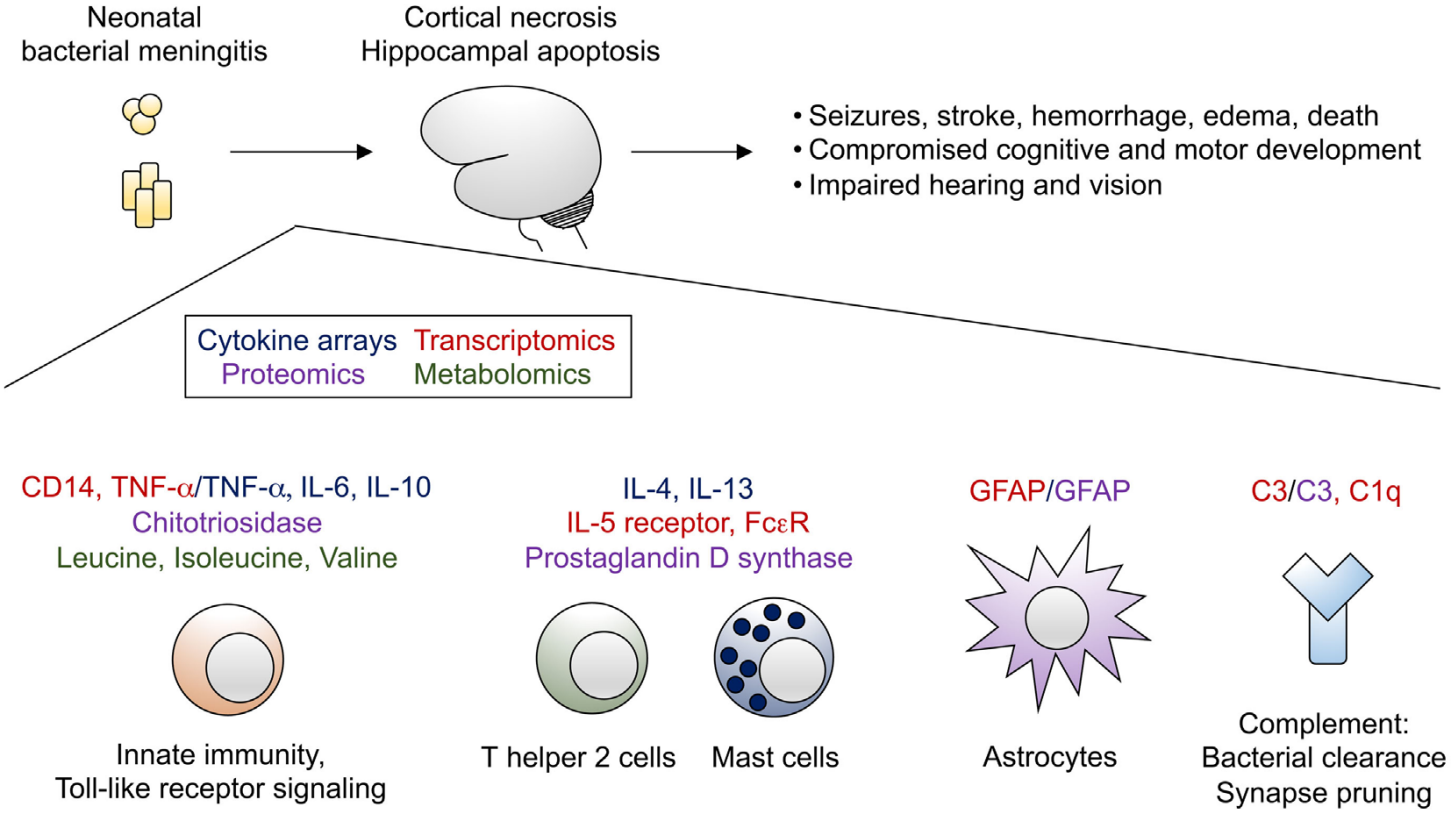
Bacterial meningitis of the neonate leads to profound brain injury, characterized by cortical necrosis and hippocampal apoptosis. Selected transcripts (highlighted in red), cytokines (highlighted in blue), other proteins (highlighted in purple), and metabolites (highlighted in green) identified from omics analyses have been integrated into a proposed model of immune activation in the setting of bacterial meningitis.

Despite improvements in neonatal intensive care and timely administration of appropriate broad-spectrum antimicrobial agents, bacterial meningitis remains a major cause of morbidity and mortality in the neonatal period. Our understanding of mechanisms of neonatal brain injury due to meningitis remains limited. Although use of cytokines as biomarkers of meningitis has not achieved sufficient accuracy to be employed in clinical practice, these data complement a wealth of omics data from humans and model organisms with bacterial meningitis that now exist. Efforts to integrate these data sets and assemble pathway maps of genes, proteins, and metabolites altered in the setting of bacterial meningitis (Figure 1) will be of great benefit to advance the field. Subsequent approaches combining omics analyses of primary human tissue (e.g., CSF) with targeted studies in animal models will be required to understand the complex interplay among the numerous infiltrating and brain-resident cell types affected by bacterial meningitis. Such work can drive development of novel, rapid diagnostics and adjunctive therapies that may prevent the devastating, lifelong sequelae of bacterial meningitis in infants.

## Author Contributions

SG, LS, and MH jointly conceived, wrote, and critically revised the manuscript.

## Conflict of Interest Statement

The authors declare that the research was conducted in the absence of any commercial or financial relationships that could be construed as a potential conflict of interest.
